# Biology, Ecology and Management of the Invasive Navua Sedge (*Cyperus aromaticus*)—A Global Review

**DOI:** 10.3390/plants10091851

**Published:** 2021-09-07

**Authors:** Boyang Shi, Olusegun O. Osunkoya, Aakansha Chadha, Singarayer K. Florentine, Kunjithapatham Dhileepan

**Affiliations:** 1 Biosecurity Queensland, Queensland Department of Agriculture and Fisheries, Boggo Road, Dutton Park, QLD 4102, Australia; olusegun.osunkoya@daf.qld.gov.au (O.O.O.); Kunjithapatham.Dhileepan@daf.qld.gov.au (K.D.); 2 Future Regions Research Centre, School of Science, Psychology and Sport, Federation University Australia, Mount Helen, VIC 3350, Australia; aakansha.chadha@gmail.com (A.C.); S.florentine@federation.edu.au (S.K.F.)

**Keywords:** Navua sedge, invasive alien species, integrated weed management strategies

## Abstract

Navua sedge (*Cyperus aromaticus* (Ridley) Mattf. & Kukenth) is an invasive perennial sedge, native to tropical Africa, which is threatening many natural ecosystems and agroecosystems, especially in northern Queensland, Australia. Crop and pasture production have been impacted by Navua sedge and it is also directly causing reductions in dairy and beef production in affected regions. This review documents the biology, ecology and potential management options to minimise the spread and impact of Navua sedge. The weed reproduces both sexually (seeds) and vegetatively (via underground rhizomes). Its tiny seeds can be spread easily via wind, water, vehicles, farm machinery and animals, whilst the rhizomes assist with establishment of dense stands. The CLIMEX model (which uses distribution and climate data in native and novel ranges) indicates that in Australia, Navua sedge has the potential to spread further within Queensland and into the Northern Territory, New South Wales and Victoria. Several management strategies, including mechanical, chemical and agronomic methods, and their integration will have to be used to minimise agricultural production losses caused by Navua sedge, but most of these methods are currently either ineffective or uneconomical when used alone. Other management approaches, including biological control and mycoherbicides, are currently being explored. We conclude that a better understanding of the interaction of its physiological processes, ecological patterns and genetic diversity across a range of conditions found in the invaded and native habitats will help to contribute to and provide more effective integrated management approaches for Navua sedge.

## 1. Introduction

Navua sedge (*Cyperus aromaticus* (Ridley) Mattf. & Kukenth) is native to equatorial African, the Seychelles, Mauritius and Madagascar and has been declared as a weed in Singapore and several island nations of the Pacific including French Polynesia and Fiji [1,2,3]. Since its introduction in the 1970s into the wet tropical regions of northern Queensland in Australia [4,5], Navua sedge has become an aggressive perennial, causing negative impacts in the beef, dairy and cropping industries [4,5,6]. It has spread along many roadsides and into grazing pastures across the wet tropics in northern Queensland. In this latter respect, Navua sedge has a low nutrient value and, as a consequence, cattle do not like to feed on them, thus contributing to its unfettered spread [6,7]. Navua sedge has been recognised as a problematic invasive weed for decades in pastures, roadsides, lawns, and crops, including banana (*Musa acuminata* Colla), sweet potato (*Ipomoea batatas* L.) and sugarcane (*Saccharum officinarum* L.) farms of wet tropical areas within Australia [6].

Of concern is that Navua sedge has serious negative effects on the biodiversity of native grasslands in Australia and other places around the world [1,4,8,9]. In Fiji, it was first found around Navua town in Viti Levu in 1933, subsequently spreading into wetter parts and becoming a major weed in Viti Levu, the largest island in Fiji [10]. It has subsequently spread into wetter parts of the island, becoming a major weed with up to 55% of the dairy pasture areas in Fiji now being covered by Navua sedge [10]. It has also been found widely in grazing areas, roadsides, lawns and turf, coconut plantations and cropping land [7]. Consequently, it was declared as a noxious weed in 1958 in Fiji [7]. At the same time, in Malaysia Navua sedge has been found to be the most abundant and dominant weed covering more than 50% of the turfgrass sites surveyed, including athletic fields, golf course grounds and resident lawns in the Klang Valley of western Peninsular Malaysia [11,12]; it has also been found in coastal rice field regions of Sebarang Perak in West Malaysia [13]. In Queensland, Australia, Navua sedge was first recorded in Cairns in 1979, but in the same year, it was also recorded in the nearby upland town of Kuranda (~15 km apart). Thus the actual pathways/entry points of introduction into Queensland remain unknown and debatable [14]. Navua sedge is highly invasive, being able to compete aggressively with desired agricultural species [4] and is readily dispersed by both seeds (birds, wind, flood and storm) and by fragmented rhizomes. It can compete with tropical pasture species as well as harbour plant pests, diseases and rats, all of which can significantly affect crop production [2]. Presently, in Queensland, Navua sedge is not a prohibited or restricted invasive plant under the *Biosecurity Act 2014* (https://www.business.qld.gov.au/industries/farms-fishing-forestry/agriculture/land-management/health-pests-weeds-diseases/weeds-diseases/invasive-plants/other/navua-sedge; accessed on 17 August 2021).

Currently, mechanical and chemical control options are mainly used to manage Navua sedge in Australia and Fiji [4,5,6]. However, it has been found that these approaches are not sustainable due to short-term effectiveness and adverse impacts to the environment of the management tactics. For example, mechanical control methods can assist the dispersal of Navua sedge to weed-free areas through active dispersal of its seeds and unwitting transport of fragments of rhizomes. In terms of chemical treatment, there is only one registered herbicide (Sempra: halosulfuron-methyl) used to manage Navua sedge but this is apparently not effective on the subterranean rhizomes. These inherent problems suggest that additional approaches such as biological control are required, which is currently being explored. In all, it is anticipated that integrated weed management methods are likely to be required for effective management of Navua sedge. This review investigates trait attributes of Navua sedge that are contributing to its spread into novel ranges and recommends potential control methods for its management.

## 2. Taxonomy and Morphology

Navua sedge is a member of the Cyperaceae—the third largest monocotyledonous family containing approximately 5500 species [15,16]. The Cyperaceae is classified into two subfamilies Cyperoideae and Caricoideae, consisting of 12 tribes and 122 genera [17]. Species within this family are difficult to distinguish due to the complex structure of their inflorescences which play an important role in their classification and phylogeny [16,18]. Within the tribe *Cypereae*, two genera are included in the *Cyperus* clade: the largest genus *Cyperus* contains approximately 950 species, whilst another smaller genus *Androtrichum* contains only two species [19]. Highlighting the complexity of this area of interest, the genus *Kyllinga* was isolated from genus *Cyperus* based on its two scaled and one flowered spikelets [20], while Navua sedge, a member of Cyperoidea subfamily, was separately identified based on its tetracyclic flowers [17]. However, molecular studies have mapped the genetic similarities in *Kyllinga* and *Cyperus*, and question their segregation into two different genera [21,22]. Further, *C. aromaticus* was previously known as *Kyllinga polyphylla* Willd. ex Kunth and *Kyllinga aromatica* Ridley, but the name *Kyllinga polyphylla* is still used in most African countries, while in Malaysia it has been recognised as Greater Kyllinga [12,23].

**Figure 1:**
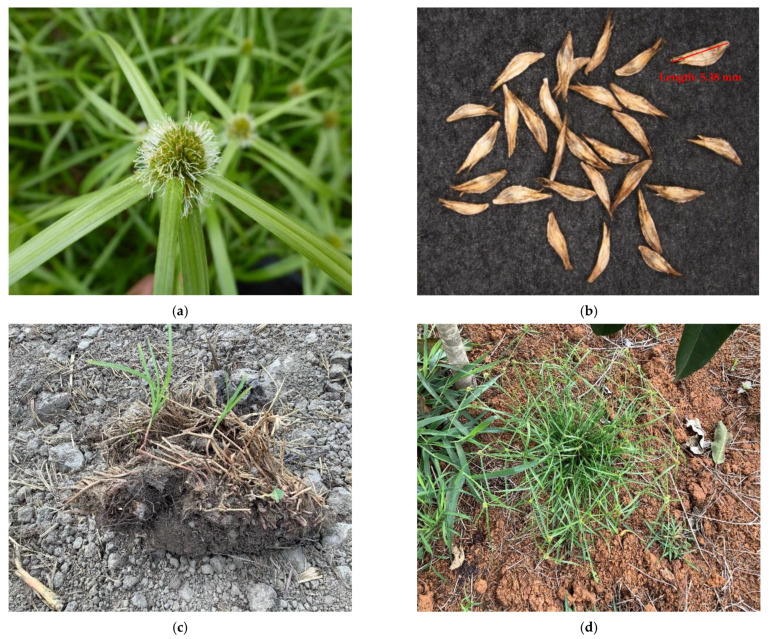
The invasive Navua sedge (*Cyperus aromaticus*); (**a**) mature flowering plants, (**b**) spikelets, (**c**) rhizomes and (**d**) mature plants in northern Queensland in Australia.

Navua sedge is a clump-forming, perennial, rhizomatous sedge, 30–60 cm tall (but occasionally up to 2 m) [9]. It possesses a robust perennial, creeping rhizome and densely set culms (stems). It has a continuously growing underground stem (rhizome) that produces shoots at regular intervals along its length [6,10]. The rhizome possesses scales about 5 mm thick, pale brown to dark purple or blackish, and less than 10 mm long (Figure 1c) [10,24]. Its culms are closely spaced and triangular in cross-section. Leaves are lanceolate, about 15 cm long and 3–5 mm wide, are glabrous and generally clustered at the base of the plant (Figure 1a). Flowers are arranged as clusters at the apex of the flower stalk, subtended by five or more leaf-like bracts (Figure 1a). The spikelets are egg-shaped, brown to black, with a hook on one end (Figure 1b). The basal part is usually covered by purplish sheaths without leaf blades, while the upper leaf-sheaths are covered with blades [24]. The inflorescence is an irregular hemispheric to globose head with a central spike and usually several smaller lateral spikes (Figure 1) [24,25].

There are different numbers of bracts of Navua sedge in its native range in west and east Africa where during a recent survey, Navua sedge with 3–7, 5–9 and 4–7 bracts have been observed in Nigeria, Tanzania and Zanzibar, respectively. However only 5–8 bracts have been found in the field in Australia. Furthermore, there are a few morphologically similar plant species to Navua sedge, such as *C. brevifolius* (Rottb.) Endl. ex Hassk., *C. erectus* (Schumach.) Mattf. & Kük., *C. melanospermus* (Nees) Valck.Sur. and *C. sesquiflorus* (Torr.) Mattf. & Kuek. [24], which necessitated careful identification during field surveys for documenting the extent of distribution, prospecting of biological control agents and plant materials collections. *C. erectus* is currently not present in Australia; key differences between *C. melanospermus* and *C. aromaticus* are that the former has fewer leaf blades and a golden inflorescence [24]. *C. brevifolius* also has a horizontal creeping rhizome and frequently evidences of a cross-like arrangement of some of the involucre bracts. In addition, its inflorescence is a solitary globose to ovate head with a greenish colour that, on maturity, becomes brown [24]. *C. sesquiflorus* has crowded culms and a short creeping rhizome, and its inflorescence is often a single ovate spike, occasionally showing a few small lateral spikes next to the central spike [24].

## 3. Life Cycle

**Figure 2:**
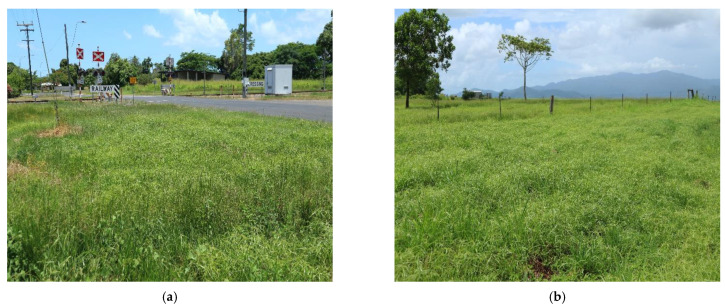

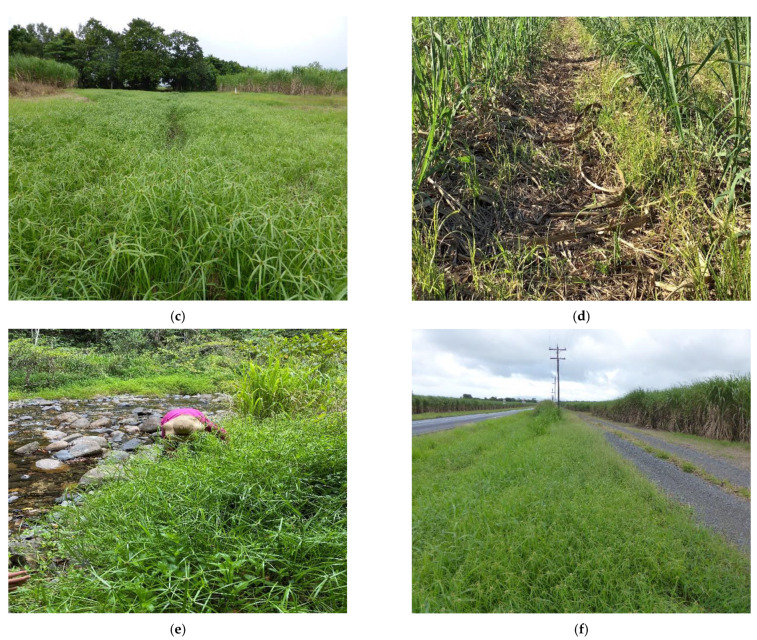
The invasion of Navua sedge (*Cyperus aromaticus*) in Australia; (**a**) near railway, (**b**) pasture land; (**c**,**d**) sugarcane farms, (**e**) along the creek, (**f**) along the roadside.

Navua sedge produces small seeds (~5 mm) from bisexual flowers which are primarily wind-pollinated [4,7]. Seeds remain dormant for a long time until suitable germination conditions prevail (8–16 h photoperiod with 15–25 °C temperature) [4]. Seeds can germinate easily from 2–3 weeks after maturity at any time of the year under suitable temperature, photoperiod and humidity conditions [2]. The rhizome system of Navua sedge is very efficient for vegetative propagation as it starts to grow within 2–3 weeks of seedling emergence [26,27]. New tillers are usually produced by secondary bud formation, whereas the third bud is responsible for branching the rhizome system [7]. Therefore, new tillers keep emerging from secondary buds of newly branched rhizomes and the plant will eventually form a dense canopy [7]. Consequently, Navua sedge can grow both from seed as well as through vegetative (rhizome) propagation, making it a very effective colonizer that is capable of forming mono-specific stands. This weed prefers to grow in wet soils with permanent moisture (Figure 2) [10]. Whereas livestock do not usually feed on this weed, cattle will eat its leaves and tillers when there is nothing else available in the field. In this situation, the quality and production of milk can be compromised [10].

## 4. Global Distribution

**Figure 3:**
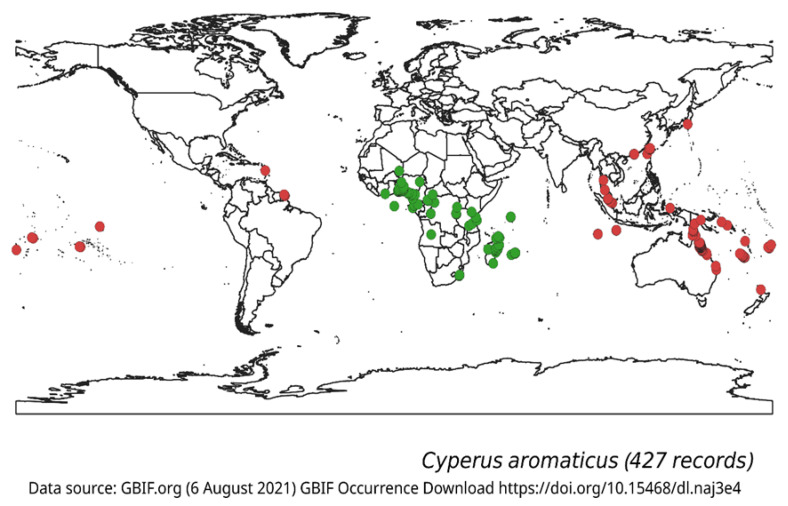
Recorded global occurrence of Navua sedge (*Cyperus aromaticus*)**.** Data of the species global occurrence were obtained from the Global Biodiversity Information Facility (2021) with a total of 427 records. Areas within the green closed circles are considered within its native range while those within the closed red circles are considered within its introduced range.

Navua sedge is a perennial sedge found in the tropics (Figure 3). It is originally from countries in equatorial Africa (Kenya, Tanzania, Congo, Gabon, Cameroon, Nigeria, Ivory Coast, Burundi) and islands in the Indian Ocean, off the coast of southeast Africa (Madagascar, Mauritius and the Seychelles; Figure 3, Table 1) [9,10]. Navua sedge has been introduced into several countries around the world including Australia, Sri Lanka, Malay Peninsula, Vanuatu, Samoa, Tahiti, the Solomon Islands and Fiji, where it has become a problematic and major weed [7,10] (Table 1).

## 5. Spread in Australia

**Figure 4:**
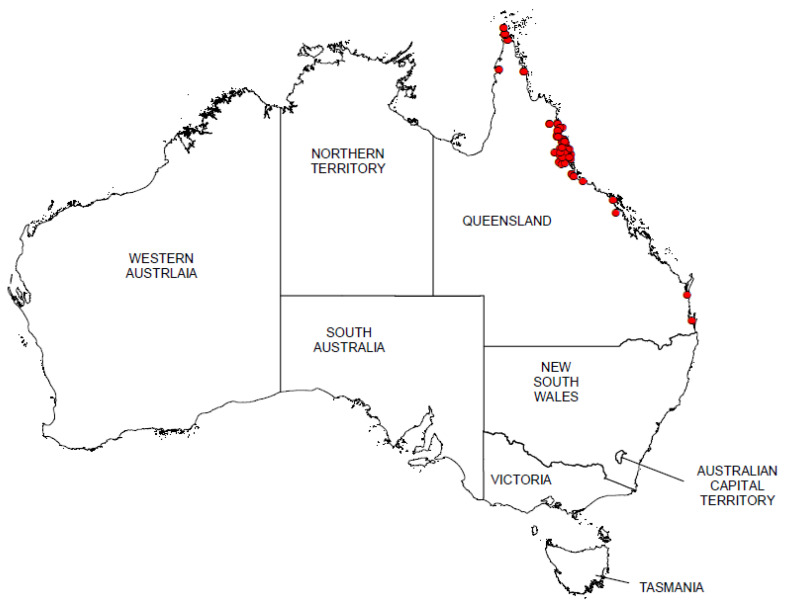
The distribution of *Cyperus aromaticus* (closed red circles) in Australia based on herbarium records (from AVH 2019).

From its initial introduction into the Cairns region of Queensland in the 1970s, Navua sedge has spread north and south of its estimated initial point of entry. The weed has become established in grazing lands and horticultural fields (such as banana, sugarcane, sweet potato, etc.) of north and far north Queensland. It is commonly found along roadsides and railway embankments from Ingham to Cape Tribulation, as well as the upland areas of the Atherton Tablelands (Figure 2). For example, it is noted that approximately 650 km of main roads and 180,000 ha of land areas are covered by Navua sedge in the Tableland regions (personal communication, Scott Morrison, Tableland regional council), where it often displaces desirable pastures species such as signal grass (*Urochloa decumbens* (Stapf) R. Webster), Rhodes grass (*Chloris gayana* Kunth) and humidicola grass (*Urochloa humidicola* (Rendle) Schweick.). Data from the Queensland herbarium and the Atlas of Living Australia suggest that Navua sedge occurs in 21 of the 664, 50 km × 50 km grids (i.e., 3.16%) of Queensland’s spatial land (Figure 4), and is ranked in the top 60% (59th out of 107) of assessed established weed species of the State in terms of spread rate and projected impact on nature conservation and agriculture [28]. In Queensland, it has been estimated that there was a short lag time of 23 years (1970–1993) prior to its population explosion [29], with a major spike in its population spread occurring between 2000 and 2010. Navua sedge dispersal rates have been estimated to be 0.2 and 0.8 grids (of 50 × 50 km grids (0.5 degree [30 min]) per year at its lag and exponential phases, respectively [29]. Nevertheless, only three of the ten regions of Queensland have so far been infested with Navua sedge, as distribution and abundance are still confined mainly to the coast. However, in view of climate change and increasing commerce, there is the potential for the weed to spread further, amongst other Queensland regions and into other States of Australia (Figure 4). State-wide, the Navua sedge invasion is categorised as stage III (requiring control and/or containment), with northern Queensland and Far North Queensland regions being in stages III and IV (requiring control-containment and/or asset protection, respectively) while minor, recent infestations (stage II category—requiring eradication and/or control) also occur in south-east Queensland.

**Figure 5:**
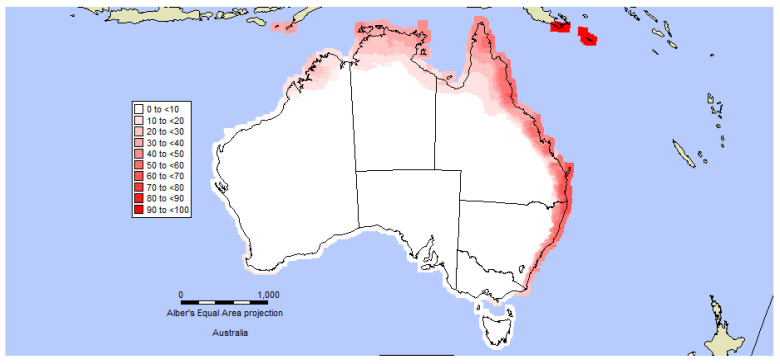
The potential distribution of *Cyperus aromaticus* in Australia as predicted by the CLIMEX model.

A species distribution model was developed using the Composite Match Index in CLIMEX to predict regions in Australia that most closely match the climate in Kenya, Tanzania and Nigeria where Navua sedge is abundant (Figure 5). The distribution data of Navua sedge were used to develop the CLIMEX model for the weed; these data were collected from different sources. Within its native range, unpublished data with 44 location points were collected from Kenya, 31 location points from Tanzania and 43 locations points recorded in Nigeria. Additional and available records on distribution of the weeds from the Australian Virtual Herbarium and the Queensland Herbarium were not used to build the model, but were used to validate the model fit [30]. The CLIMEX model utilises an annual growth index to predict the potential growth under suitable climate conditions with four stress indices (hot, cold, wet and dry) [31,32]. The primary base climatology used to build the model was the CliMond 10′ climate normal centred on 1975 (CM10_1975H_V1_1). The minimum and maximum temperature threshold were extracted from the world distribution points of the plant. The minimum and maximum temperatures were set to 13 °C and 38 °C, respectively based on the field observation and personal communication with local farmers; these values are equal to the heat stress threshold in the model. Lower and upper optimal temperature were set to 23 and 31 °C, respectively (Table 2). Navua sedge is a wet tropical species, so the lowest and highest soil moisture threshold were set to 0.3 and 2.3, respectively. Lower optimal moisture and upper optimal moisture were set to 0.7 and 1.8, respectively (Table 2). Stress value shows negative population growth [31]. The dry stress value was the same as the limiting low soil moisture and the wet stress threshold was set at the same level as the limiting high soil moisture [31,32]. An annual index of climatic suitability was calculated by the compare locations function in CLIMEX, and the Ecoclimatic Index (EI) was selected to indicate the combined population growth of potential distribution within favourable stressful conditions. The CLIMEX model was validated to some extent by using some native survey data collected from Tanzania, Kenya, Zanzibar and Nigeria. The outputs from the model suggested that Navua sedge can spread further in Queensland, and also has the potential to spread into New South Wales, Victoria and the Northern Territory (Figure 5).

## 6. Ecology

### 6.1. Typical Locations of Infestations of Navua Sedge

Navua sedge can be found along roadsides, railways, lawns, grassland, wastelands, grazed pastures, construction sites, creek banks and forests (Figure 2). Navua sedge is a perennial monocot, producing flowers and seeds throughout the year, and the seeds can also germinate at a range of temperatures, soil moisture, pH levels, salinity, radiant heat treatment, osmotic potential and light intensity [6]. It can be found in all types of soils, including black, sandy, alkaline and clay soils, but it is noted that seedling emergence decreases with increased seed burial depth [6]. As a consequence of this growth flexibility and hence ecological plasticity, these plants can potentially be found in a wide range of locations and can sprout at any time during the year. However, in Australia, it prefers to grow in the wet tropical regions where annual rainfall is above 2500 mm and where there is no distinct dry season. In areas with lower rainfall or those experiencing a periodic dry season, it is usually found in the damp, low-lying areas in pastures, drains or disturbed areas subjected to periodic flooding [33]. By comparison, Navua sedge has established in Fiji in both dry areas and wetlands, and has been reported to flourish in plantations and farms of coconut rice and sugarcane fields, as well as in pastures, lawns, and damp areas [7].

### 6.2. Seed Biology and Longevity

Seedlings of Navua sedge develop quickly and initiate flowering 7–8 weeks after emergence, with seeds requiring an additional 30 days to ripen at the flower head [4]. At the time of flowering, a new shoot is also produced on the underground stem [10,24]. This new shoot then grows as a (false) seedling, producing a flower seven weeks after emergence plus a new shoot from the underground stem [10]. This process is continually repeated and results in a rapidly spreading colony of stems growing from an interconnected underground rhizome system. Whilst the seeds can germinate at any time of the year, the highest germination rate occurs when temperatures alternate between 15 °C and 30 °C; exposure to light is also a prerequisite for germination [6]. The inflorescence heads on each shoot generally produce about 250 seeds, meaning that seed production per hectare is extremely high, in the order of ~450–550 million seeds [4]. The longevity of these seeds is greater than 15 years with a third of the seed bank viable still after five years [4]. A previous study found that more than 95% of seeds germinated in a temperature range of 15–25 °C with 8–16 h light photoperiod, and no seeds germinated under completely dark conditions [7]. Only 3% of seeds germinated under continuous light conditions [7]. The Cyperaceae family is specialised in having high seed buoyancy, a trait that helps in the spread of its propagule by air and water movement [34]. The small seeds can also hitchhike on the body of cattle, vehicles and transport equipment, thus they can be distributed into new and uninfected regions [4]. Seed can also be spread through cattle excretion occurring due to grazing on Navua sedge [7]. Furthermore, birds have been reported to gain nutrition from seeds of sedges, and hence their seeds can potentially be dispersed through their faecal matter. In this respect, these tiny seeds have less retention time in the gut of birds, allowing them to retain their structure [34,35]. In summary, it can be expected that the small seed size and structure of barbs, high viability and long dormancy of seed will contribute to the further spread to novel ranges of Navua sedge [34].

Soil pH has been shown to have minimal impact on the seed germination of Navua sedge populations collected from far north Queensland [6]. More than 85% of seeds can germinate with pH levels ranging from 4 to 10, indicating that Navua sedge seeds can easily germinate in most areas in Australia [6,36]. In terms of osmotic pressure, no seeds were able to germinate at more than 150 mM of sodium chloride concentration [6]. While soil affected by highly saline water, such as along the coastal areas, is unlikely to support seed germination, seeds will germinate in low saline water below 150 mM of sodium chloride concentration [6]. Results of the same study suggest that burning may not be effective to inhibit the seed germination of Navua sedge [6]. Seedling emergence declined dramatically when they were buried from 0.5 to 2 cm, depending on the population investigated [6]. All seeds showed the greatest emergence rate when placed on the top of soil.

### 6.3. Seed Banks

A range of 44,400 to 56,700 seeds m^−2^ were found in infested sites with approximately 200 Navua sedge plants per m^2^ of infestation areas [4]. In a previous study in Fiji, a greater number of seeds and tillers were found on a lowland paddock (45,600 seeds and 182 tillers per m^2^) compared to a hillside paddock (20,800 seeds and 83 tillers per m^2^) [7]. Half of these seeds were located in the top 2 cm of soils [4]. This trend is similar to a recent finding in infested soils of coastal lowland and uplands in far north Queensland (Osunkoya, OO, unpublished data).

### 6.4. Allelopathic Interaction

A recent study of Navua sedge has showed the possible presence of allelochemicals [3]. The number of germinating seeds of Signal grass (*Brachiaria decumbens*) and Setaria (*Setaria sphacelata*) (both are pasture grasses) were extremely low when exposed to leachates of root and culm materials of Navua sedge [3], but there was not any significant difference between culm and root leachates on the germination rate of seeds of the test species [3].

## 7. Impacts

### 7.1. Beneficial Impact

Some *Cyperus* spp., including *C. aromaticus*, have been found to be a natural source of insect juvenile hormone III, which is used as a bioinsecticide for pests such as mosquitoes [8]. However, a challenge is that juvenile hormone III cannot be reliably extracted from the plant, though it can be routinely extracted in vitro from cell culture [37]. Wild plants are therefore unlikely to be necessary for any production of this hormone and thus no beneficial aspects of Navua sedge are currently recorded in Australia.

### 7.2. Detrimental Impact

Currently, Navua sedge is neither a restricted nor prohibited plant in Queensland or any other State or Territory in Australia; notwithstanding, it is widespread in wet tropical areas of Queensland [4,6]. The sedge is unpalatable, and can form dense stands, by replacing palatable tropical pasture species. Over 1000 beef producers, dairy farmers and hay producers in the wet tropical region of north Queensland, including Atherton, Cairns, Daintree and Innisfail Regions are impacted by Navua sedge. Beef cattle and dairy farmers in these areas regard Navua sedge as a weed of major concern, since its presence results in productivity losses. Losses are caused by reduced pasture yield, reduced carrying capacity, and increased weed management costs. For the latter, chemical residues and grazing withholding (spelling) periods are of particular concern [30].

As noted earlier, Navua sedge is recorded as an important weed and widespread in Fiji, French Polynesia and Western Samoa (Figure 3) [38]. It is a declared noxious weed in Fiji. It has been found in rice, pineapple, watermelon and vegetables growing areas [39] and has been reported to have potential to enter the United States as a contaminant of grass seed and to become a weed of pastures and natural areas in any tropical region, an observation which has particular importance for Australia. It is not readily grazed by livestock and when established soon dominates a pasture, and thus reducing yield including milk, butterfat and beef production [4,40].

## 8. Management

Possible management options of Navua sedge must incorporate an understanding of the biological traits (spread patterns, abundance and ecology) of the species itself. Control options will vary, ranging from mechanical, chemical and use of biological control, to integrated weed management. Integrated weed management includes the use of several control approaches, combined in a strategy to manage a selected weed in a sustainable, cost-effective and environmentally benign manner [41,42].

### 8.1. Mechanical Control

Mechanical control of large pasture infestations has been achieved using heavy rollers at monthly intervals to repeatedly break the stems at ground level and allow other grasses to out-compete the sedge [10]. Four consecutive monthly crushing applications were shown to be able to control 42% of the original Navua sedge population [10]. Clearly, the use of rollers is impractical in hilly country and generally mechanical control methods are not a long-term solution as it requires repeated applications. Regular slashing and mowing of Navua sedge along roadsides, while aesthetically pleasing, can easily spread the light, tiny seeds to other landscapes and regions, and hence are not recommended for managing Navua sedge.

### 8.2. Chemical Control

In Australia, herbicide control is limited to the use of Sempra (halosulfuron-methyl) which is listed under the minor use permit PER80065 (http://permits.apvma.gov.au/PER80065.PDF, accessed on 3 September 2021) [5]. Herbicide must be applied every 8–10 weeks during February to October to minimise Navua sedge seed being added to the soil seed bank [4,5]. However, treatment costs are high, running at about $435.89/ha/year (personal communication, Scott Morrison, Tableland regional council). Additionally, the herbicide does not act directly on the subterranean structure of the plant and hence may not be cost effective and/or sustainable.

### 8.3. Biological Control

Biological control, which has generally been found to be the most cost-effective and long-term management option for many introduced weeds [43], has not been explored with Navua sedge to date. It is anticipated that biological control in Australia will help to greatly reduce Navua sedge vigour and seed production and assist in minimising its impact and spread. A biological control program that is effective will also significantly lower the need for herbicide applications. As a prerequisite to commencing a weed biological control program, Navua sedge has been nominated and approved as a candidate for weed biological control in Australia in 2020.

Further, in response to stakeholders’ demand, a study to search for specialist natural enemies as biological control agents for Navua sedge in its native range has commenced. As indicated earlier, the native range of Navua sedge includes countries in East and West Africa. Based on logistics and safety, Kenya, Tanzania and Nigeria were identified as prospective areas for native range surveys. These surveys have identified a new smut fungus (*Cintractia kyllingae* [44]) which attacks flower heads and seeds and a rust fungus attacking leaves and stems (*Uredo kyllingae-erectae*) as being promising biological control agents [45]. It is recognised that for effective biocontrol, multiple agents, targeting different parts of the sedge are needed, and relevant host specificity tests for the smut fungus are currently in progress at CABI-UK.

### 8.4. Cultural Control

Competition studies using different pasture species have been undertaken in Australia. The studies compared the impact of competition from signal grass (*Brachiaria decumbens* Stapf) and setaria grass (*Setaria sphacelata* (Schumach.) Stapf & C.E. Hubb.) under glasshouse conditions [3]. Results showed that setaria grass is a better competitor for Navua sedge than signal grass [3]. Furthermore, another recent competition study using the pasture species Rhodes grass (*Chloris gayana* Kunth) and humidicola grass (*Brachiaria humidicola* (Rendle) Schweick under two different field moisture capacity levels (50% and 100%) showed that Rhodes grass resulted in greater suppression of Navua sedge than humidicola grass, but there were no significant differences between the two different field moisture capacity levels, indicating that moisture level is not a factor influencing competition in this particular case [46]. In a separate study, the same two test species (Rhodes grass and humidicola grass) gave the same result [47]. In addition, a “gap” series trial using two different ages (one- and two-months-old) of two tropical pasture species (signal grass and setaria grass) with different distances (0, 2.5, 5, 10, 15 and 20 cm) to the central single seedling of Navua sedge was conducted [3]. A reduced number of tillers and lower biomass for Navua sedge seedling were produced when grown together with Setaria grass compared to Signal grass [3]. Both older grasses showed more inhibition of Navua sedge growth than younger plants [3]. The greatest suppression of Navua sedge growth was discovered at 2.5 cm gap size, with the smaller gaps from both grasses to Navua sedge showing better suppression of the growth of Navua sedge [3].

### 8.5. Integrated Management

In summary, previous studies suggest that Navua sedge will be poorly managed if a single approach in isolation is used and hence, integrated management using a range of approaches is recommended [4,10]. This integrated management should also involve local groups (farmers, local government pest/biosecurity officers, parks and wildlife rangers, and the wider community) who will inevitably play important roles in the successful development and implementation of integrated management strategies for Navua sedge.

## 9. Management Challenges

The rhizomes of Navua sedge have a dual function, acting both as organs of survival and reproduction. In this respect, they store carbohydrates and produce vegetative buds. Indeed, one of the primary reasons that perennial weeds like Navua sedge are so persistent in nature is their ability to store significant reserves in their underground organs [48]. Resprouting, regrowth potential and aboveground phenological development are strongly correlated with the total non-structural carbohydrate content of the rhizomes, making it important to understand the biological traits of these structures. Vegetative buds present on the rhizome constantly add tillers to the plant which, in turn, produce seeds. In addition, these buds also provide plants with a safety net for regrowth or reproduction if the actively growing portion of the plant dies [7]. Another challenge in managing Navua sedge is that not all the vegetative buds are in the same developmental stage, with some buds being dormant due to restrictions in bud activity [7]. With regards to management, the ability to release and kill all nodes will be most beneficial as it will eliminate the regenerative ability of the rhizomes. Hence, knowledge about the conditions that induce and break dormancy in the buds of rhizomes will be key in designing future management strategies.

As Navua sedge produces a vast number of seeds which are known to persist for longer than 15 years in the soil, due consideration should also be given to reduce the input of seeds into the soil seed bank and to exhaust the soil-stored seedbank, since new infestations emerging from seeds can produce high plant densities [4]. Recent work has indicated that it is comparatively easy to control the emerged aboveground shoots of Navua sedge, either by mechanical control like crushing, or by using herbicides like Sempra [4]. However, it is much more challenging to ensure translocation of an adequate amount of herbicide throughout the extensive underground rhizome system. If the buds present on the rhizome are not affected by the herbicide, they have the capacity to sprout and regenerate and continue the life cycle. Hence, it is clearly important to limit the production of seeds as well as reproduction/propagation via rhizomes simultaneously.

The effect of 17 herbicides was examined in field trials, and they indicated that only six herbicides were effective against Navua sedge [4]. These herbicides provided above 90% control at very high rates, but this could cause environmental problems such as their lack of selectivity, movement off-site, and persistence in the soil [4]. The use of non-selective herbicides may also damage other pasture plants, thereby reducing the pasture cover which, in turn, would then create opportunities for Navua sedge to spread. Only one active ingredient (halosulfuron) is currently being used in Australia and this single management strategy is not sufficient for the control of Navua sedge [5]. Pastures can only be sprayed with Sempra twice per year and there is a grazing withholding (spelling) period of 10 weeks, which does align with stakeholders’ production schedules. Furthermore, despite the fundamental role of rhizomes in driving the population dynamics of Navua sedge, Sempra has only been found to target the aboveground biomass, suggesting that current chemical options to control the rhizomes of Navua sedge are very limited. More research is required on the absorption, translocation, and metabolism of herbicides in Navua sedge to address control of underground reproductive components.

Another challenge faced in the control of Navua sedge is that the plants growing from rhizomes do not need an initial establishment period. They can grow quickly, thus establishing in situ competition to economically valuable crops or pastures, which require some time to establish. Whilst it is important to continue to suppress rhizome development and to reduce the seasonal aboveground biomass to control the spread via seeds, it is equally important to ensure that Navua sedge management should (i) focus on long-term control of Navua sedge using integrated weed management strategies, (ii) be arranged in a cost-effective way to be affordable to farmers and other stakeholders (e.g., Weed and Natural Resources management agencies), (ii) be organised in a manner which causes minimum adverse environmental side effects, (iii) be introduced in a way which assures stakeholder’s acceptance of the control practices, (iv) be able to be maintained in a sustainable manner that minimises recurrent costs and promotes ecological equilibrium, and (v) be designed to allow practical implementation from a paddock scale to landscape scale.

## 10. Conclusions

The general concern is that Navua sedge is becoming a problematic weed in northern Queensland; currently, there are limited approaches available for its management. Whilst mechanical and chemical methods are the dominant control options, these are not recommended due to the inherent risk of catalysing further spread and high costs of control. We believe that other methods, including biological control, will need to be investigated in order to complement chemical and physical methods, for effective management of Navua sedge. The use of vigorous and competitive fodder plants to suppress the growth of Navua sedge can be another method worth investigating. There are knowledge gaps that have been highlighted in the review, including several aspects of Navua sedge’s biology and ecology, such as genetic diversity in the native and invaded ranges, seed banks dynamics, allelopathic interference and phytotoxicity are still missing or unpublished. In particular, genetic studies would provide valuable information on the exact origin of the Navua sedge populations invading Australia, which should in turn facilitate the efforts to find effective biological control agents.

## Figures and Tables

**Table 1:** The countries within Africa where Navua sedge is thought to be native and the countries in Asia, Oceania and America where Navua sedge has been introduced.

Africa (Native)	Asia (Introduced)	Oceania (Introduced)	America (Introduced)
Angola	British Indian Ocean Territory	Australia	Martinique
Burundi	Brunei	Fiji	French Guiana
Cameroon	China	French Polynesia	
Central African Republic	Hong Kong	New Caledonia	
Comoros	India	New Zealand	
Congo	Malaysia	Samoa	
Gabon	Sri Lanka	Solomon Islands	
Ghana	Taiwan		
Guinea	Thailand		
Kenya	Vietnam		
Madagascar			
Mauritius			
Nigeria			
Reunion			
Seychelles			
Somalia			
South Africa			
Tanzania			
Togo			
Uganda			

**Table 2:** The indices and parameters used in the development of the CLIMEX model for *Acaciothrips ebneri* based on its native range distribution.

Index	CLIMEX Parameters	Value
Temperature	Limiting low temperature (DV0)	13 °C
	Lower optimal temperature (DV1)	23 °C
	Upper optimal temperature (DV2)	31 °C
	Limiting high temperature (DV3)	38 °C
Moisture	Limiting low moisture (SM0)	0.3 ^a^
	Lower optimal moisture (SM1)	0.7 ^a^
	Upper optimal moisture (SM2)	1.8 ^a^
	Limiting high moisture (SM3)	2.3 ^a^
Cold stress	Cold stress temperature threshold(TTCS)	5 °C
	Cold stress temperature rate (THCS)	−0.01 week^−1^
	Cold stress degree-day threshold DTCS)	0 °C days
	Cold-stress degree-day rate (DHCS)	0 week^−1^
Heat stress	Heat stress temperature threshold (TTHS)	38 °C
	Heat stress temperature rate (THHS)	0.003 week^−1^
	Heat stress degree-day threshold	0 °C days
	Heat stress degree-day rate	0 week^−1^
Dry stress	Dry stress threshold (SMDS)	0.3 ^a^
	Dry stress rate (HDS)	−0.002 week^−1^
Wet stress	Wet stress threshold (SMWS)	2.3 ^a^
	Wet stress rate (HWS)	0.005 week^−1^

^a^ Values without units are a dimensionless proportion.

## Data Availability

Not applicable.
